# Preparing of Highly Conductive Patterns on Flexible Substrates by Screen Printing of Silver Nanoparticles with Different Size Distribution

**DOI:** 10.1186/s11671-016-1640-1

**Published:** 2016-09-20

**Authors:** Jin Ding, Jun Liu, Qingyong Tian, Zhaohui Wu, Weijing Yao, Zhigao Dai, Li Liu, Wei Wu

**Affiliations:** 1 Laboratory of Printable Functional Nanomaterials and Printed Electronics, School of Printing and Packaging, Wuhan University, Wuhan, 430072 People’s Republic of China; 2 Key Laboratory of Artificial Micro- and Nano-structures of Ministry of Education, School of Physics and Technology, Wuhan University, Wuhan, 430072 People’s Republic of China; 3 Shenzhen Research Institute of Wuhan University, Shenzhen, 518057 People’s Republic of China

**Keywords:** Ag nanoparticles, Size distribution, Electrical resistivity, Screen printing, Printed electronics

## Abstract

A facile one-step polyol method is employed to synthesize the Ag nanoparticles (NPs) in large scale. The Ag NPs with different average diameter (from 52 to 120 nm) and particle size distribution are prepared by changing the mass ratio of AgNO_3_ and PVP. Furthermore, the as-obtained Ag NPs are prepared as conductive inks, which could be screen printed on various flexible substrates and formed as conductive patterns after sintering treatment. During the reaction process, PVP is used as the capping reagent for preventing the agglomeration of Ag NPs, and the influence of the mass ratio of AgNO_3_ and PVP to the size distribution of Ag NPs is investigated. The results of electronic properties reveal that the conductivity of printed patterns is highly dependent on the size distribution of as-obtained Ag NPs. Among all the samples, the optimal conductivity is obtained when the mass ratio of AgNO_3_ and PVP is 1:0.4. Subsequently, the sintering time and temperature are further investigated for obtaining the best conductivity; the optimal electrical resistivity value of 3.83 μΩ · cm is achieved at 160 °C for 75 min, which is close to the resistivity value of the bulk silver (1.58 μΩ · cm). Significantly, there are many potential advantages in printed electronics applications because of the as-synthesized Ag NPs with a low sintering temperature and low electrical resistivity.

**Electronic supplementary material:**

The online version of this article (doi:10.1186/s11671-016-1640-1) contains supplementary material, which is available to authorized users.

## Background

Printed electronics represents a promising research field for flexible electronics because of the advantages of mass production, low cost, and environment friendly, which receives growing interesting in recent years [1–5]. Various printing technologies are proposed to facilitate more rapid development of printed electronics, such as inkjet printing, gravure printing, offset printing, and screen printing﻿,﻿ etc [6–12]. Among them, screen printing route is often considered as the most suitable candidate for printed electronics because of its intrinsic simplicity, affordability, high speed, mass production capability, and versatility [13]. Moreover, the screen printing is also a popular technique for printing on various rigid or flexible substrates [14]. It is noteworthy that the conductive ink is the critical factor to fabricate the desired patterns with required conductivity and quality in screen-printed electronics [1, 15]. Recently, many nanomaterials are developed and used as conductive inks, including metallic nanoparticles (NPs), conductive polymers [16], graphene [17], carbon nanotube (CNT) [18, 19], etc. [20]. The conductive polymers, graphene, and CNT as conductive ink are widely used to print integrated circuits [21]. However, the conductivity of such inks still has a room to improve and elevate [22]. Therefore, ink formulations based on metallic NPs are selected as the strong candidate because of its desirable conductivity [23–25]. Currently, silver is the most reported material for conductive ink and also the most utilized in industrial applications.

It has been reported that the average diameter and particle size distribution of nanomaterials are closely related to the properties, such as optical, magnetic, and electronic properties [26–29]. For example, Jiang and co-workers prepared Ag NPs with various sizes by citrate reduction method. When the size of the Ag NPs is decreased, the corresponding photoluminescence spectra shifted to higher energies, which reveal that the optical property of Ag NPs is dependent on particle size [30]. However, the relationship between the size distribution and the electrical properties of the synthetic Ag NPs is rarely studied in detail, especially in printed patterns and printed devices.

Additionally, the use of Ag NP-based inks requires post-print sintering of the printed layers to form electrically networks and high conductive layers, and the sintering step can remove the organic component of inks [31, 32]. For example, Lee and co-workers printed Ag NP-based pastes with 50–100 nm in size on alumina substrates by screen printing, the optimal electrical resistivity of the film is 4.11 μΩ · cm when the printed film was sintered at 450 °C for 15 min [33]. The obtained value of electrical resistivity may be compatible for various electronic devices, but the high sintering temperature of 450 °C is not useful for flexible printed electronics. This is because most of flexible substrates (such as paper, polyethylene terephthalate (PET), and polyvinyl chloride (PVC)) cannot resist to this high sintering temperature [34–36]. Cho and co-workers printed silver lines by screen printing with size around 20-nm Ag NPs and then a low temperature of 200 °C was chosen to sinter for 1 h. The lowest resistivity value of the synthesized inks is 33 μΩ · cm [37]. The sintering temperature is low enough for flexible substrates, but the resistivity values of the synthesized inks still need to improve [32]. Obviously, the improvement of electrical property at low sintering temperature is the eventual purpose for the as-synthesized Ag NP-based inks.

Herein, the Ag NPs with different size distribution are prepared through a one-step, high-effect, and high-reliability polyol method. The relationship between the size distribution and the electronics properties of printed Ag NPs layers are demonstrated. Furthermore, in order to obtaining high conductivity, the different sintering temperature and time of these final Ag NP-based patterns are further studied. Then, the SEM technique is applied to investigate the sintering mechanism of the as-prepared designed patterns.

## Methods

### Materials and Chemicals

Silver nitrate (AgNO_3_, 99.0 %), anhydrous alcohol (CH_3_CH_2_OH), and ethylene glycol (EG, 99.0 %) were purchased from Sinopharm Chemical Reagent Co., Ltd. Polyvinylpyrrolidone (PVP, MW = 10,000) was purchased from Aladdin Chemistry Co., Ltd. All chemicals were analytical grade and used as received without further purification. The high temperature-resistant PET (~210 °C, the thickness is 0.3 mm) and A4 paper were used as flexible substrates. Deionized water got from a Millipore system (ρ = 18.2 MΩ) was used in through experiments.

### Synthesis of Ag NPs

The Ag NPs are prepared by the reduction of AgNO_3_ in EG solution in the presence of PVP. Typically, 0.531 g of AgNO_3_ and 0.531 g of PVP were dissolved in 25 mL of EG. Then, the mixture was heated up to 120 °C in an oil bath with vigorous magnetic stirring for 30 min. The obtained products were centrifuged at 12,000 rpm for 5 min and washed several times by ethanol. The Ag NPs with different morphology and size distribution are adjusted by the mass ratio of AgNO_3_ and PVP, which are named as S1–S6, respectively. Finally, the as-obtained Ag NPs were re-dispersed in ethanol for further characterization and application.

### Deposition of Ag NP-Based Conductive Inks on PET Substrate

The Ag NP-based conductive inks were prepared by directly dispersing in ethanol, the weight percentage of Ag NP-based conductive inks is *ca*. 70 %, and deposited on the PET substrates. Finally, the patterns were sintered at 160 °C for 75 min in a drying oven. Additionally, different kinds of patterns including arrays, lines, and tags are screen printed. Here, the mesh count of the used screen printing plates is 300 fibers per centimeter. During the screen printing process, the as-prepared Ag inks are through the screen mesh and transferred to substrate under the pressure of squeegee.

### Characterization

The morphology analysis of the as-synthesized Ag NPs and the printed patterns were performed with a field emission scanning electron micrograph (FE-SEM) (Hitachi S-4800). The ultraviolet-visible (UV-vis) absorption spectra were recorded on a Shimadzu 2550 spectrophotometer. The X-ray diffraction (XRD) patterns of the samples were characterized on an X-ray diffractometer (PANalytical X’Pert Pro) with Cu Kα radiation operated at 40 kV and 40 mA at a scan rate of 0.05° 2θ s^−1^.

### Electrical Performance Test

The electrical performance experiments are performed by using the four-point probe resistance tester (FP-001), then the electrical conductivity of the printed patterns is measured. Each sample is tested for ten times. Then, the average value of each sample is used to calculate the resistivity and the electrical conductivity. The bulk resistivity is calculated firstly by the following equation:

1 $$ \rho ={\mathrm{R}}_{\square}\times W={\mathrm{R}}_{\mathrm{X}}\times F\left(D/S\right)\times F\left(W/S\right)\times {F}_{\mathrm{SP}}\times W $$

where *ρ* is the electrical resistivity (μΩ · cm) and R_□_ and R_X_ are the sheet resistance and instrument measuring resistance of these as-prepared Ag-based patterns, respectively. *W* and *D* are the thickness and diameter of the pattern, and *F* (*D/S*) and *F* (*W/S*) are the constants. *F*_SP_ is the probe spacing correction factor. The bulk electrical conductivity of the Ag patterns is the reciprocal of electrical resistivity:

2 $$ \sigma =1/\rho $$

where σ is the electrical conductivity.

## Results and Discussion

**Fig. 1:**
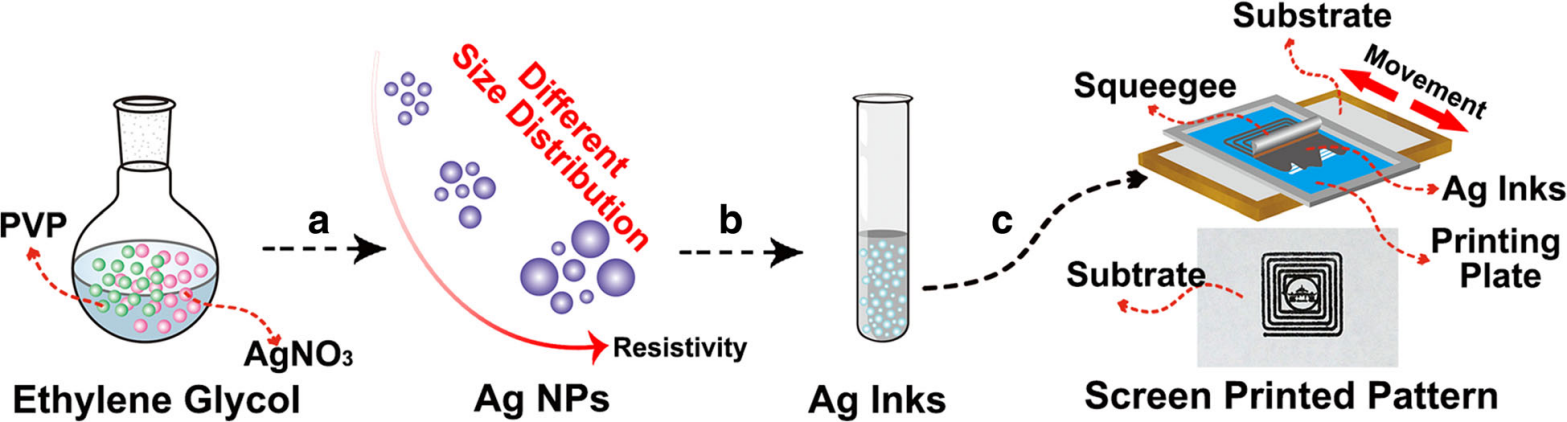
Schematic illustration of the preparation process of Ag NP-based conductive inks and screen printing conductive patterns. **a** The controlled mass ratio of AgNO_3_ and PVP for obtaining of Ag NPs with different size distribution. **b** The as-synthesized Ag NPs are dispersed into ethanol for the formation of Ag inks. **c** Screen printing of Ag inks on different substrates with designable patterns

As shown in Fig. 1, Ag NPs are firstly synthesized by reducing of AgNO_3_ in EG solution and using PVP as a capping agent. The size distribution of Ag NPs is altered by adjusting the mass ratio of AgNO_3_ and PVP. Then, the obtained Ag NPs are employed as conductive inks by simply dispersing into ethanol for screen printing. During the screen printing process, the Ag inks are spread onto the upper design pattern of plates. Finally, the designed Ag patterns are obtained after printing.

**Fig. 2:**
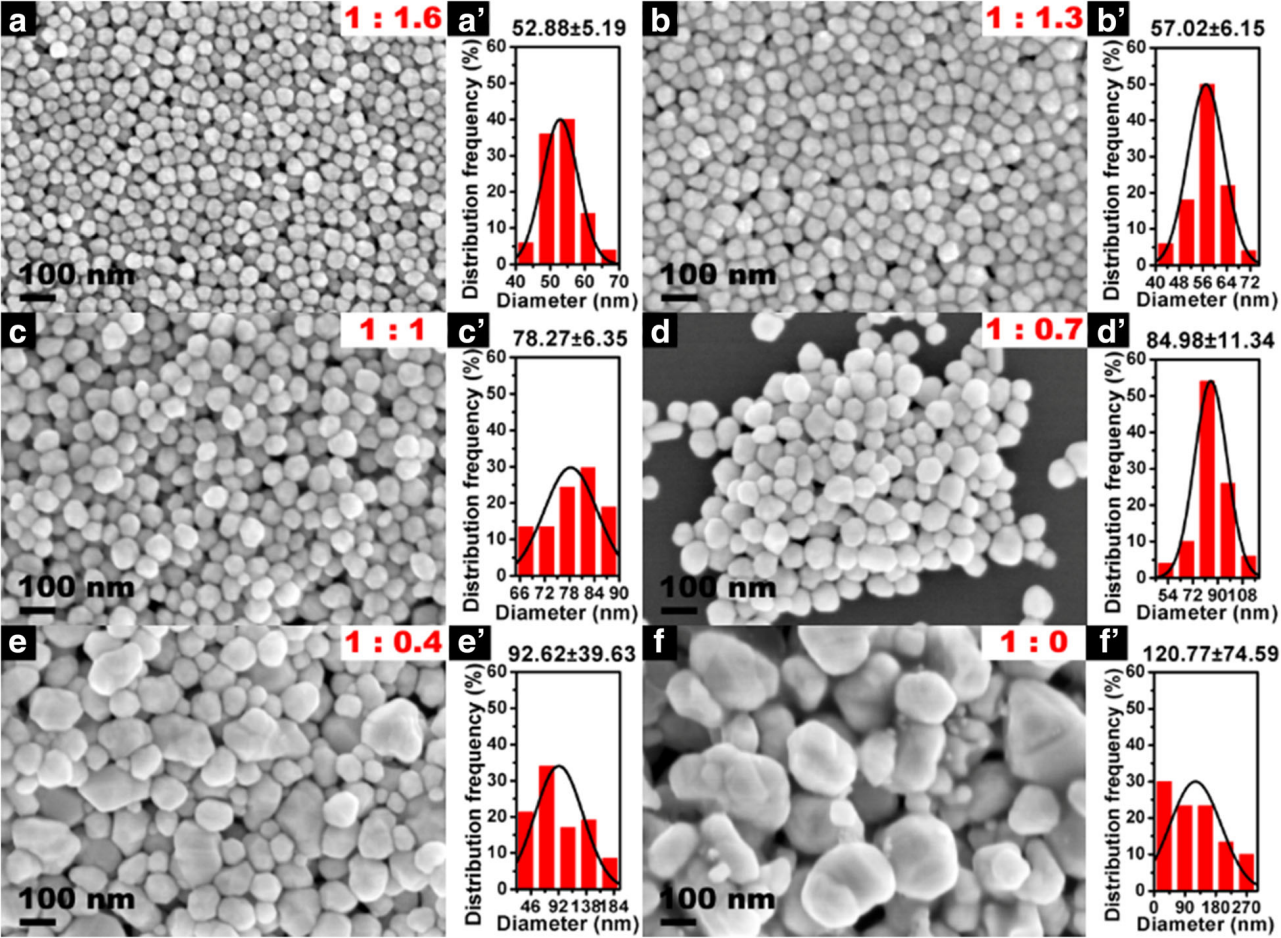
Representative SEM images of the as-synthesized Ag NPs with different mass ratio of AgNO_3_ and PVP. **a** Sample S1. 1:1.6. **b** S2, 1:1.3. **c** S3, 1:1. **d** S4, 1:0.7. **e** S5, 1:0.4. **f** S6, 1:0. **aˊ**–**fˊ** The corresponding histogram of particle size distribution (The result was statistically analyzed by JEOL Smile View software with more than 100 resoluble particles)

The SEM images and corresponding size distribution of the as-obtained Ag NPs are shown in Fig. 2. Clearly, uniform Ag NPs with an average diameter of 52.88 nm are produced when the mass ratio of AgNO_3_ and PVP at 1:1.6 (Fig. 2a and 2aˊ) is fixed, and the similar uniform Ag NPs with average diameter of 57.02 nm are also obtained when the mass ratio of AgNO_3_ and PVP to 1:1.3 (Fig. 2b and 2bˊ) is increased. However, when the mass ratio of AgNO_3_ and PVP from 1:1 to 1:0 (no PVP in reaction) is further increased, the mean diameter and standard deviation of particle size are significantly increased (Fig. 2c–f and 2cˊ–fˊ), indicating that the PVP plays an important role in regulating the morphology and size distribution of Ag NPs. Generally, PVP is a common stabilizer or capping agent against the aggregation among NPs because of the strong affinity between the preformed NP surfaces and oxygen and nitrogen atoms in the pyrrolidone unit in the colloid synthesis process [38]. With the introduction of PVP, silver ions or NPs would coordinate with N or O in PVP, and a covered layer would generate on the surface of the Ag NPs [39, 40]. This layer inhibited the growth and agglomeration of the NPs. The size of Ag NPs will become smaller and the particle size distribution of Ag NPs will be more uniform when the amount of PVP is elevated [41]. Therefore, the different size of Ag NPs could be obtained by adjusting the amount of PVP.

**Fig. 3:**
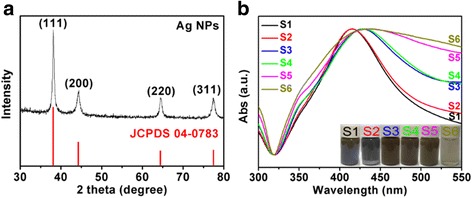
**a** Representative XRD patterns of as-prepared Ag NPs. **b** UV-vis spectra of as-prepared Ag NPs (S1 to S6) and the corresponding photographs

The structure of the synthesized Ag NPs (S1) is characterized by XRD (Fig. 3a). Clear diffraction peaks at 38 °, 44.26 °, 64.40 °, and 77.32 ° are observed, confirming that the product is cubic-phased Ag (JCPDS 04-0783). Furthermore, there is no other diffraction peaks are found, indicating that the as-prepared sample is highly purified Ag NPs. Additionally, the optical properties of the prepared Ag NPs with different size distribution are also investigated through the UV-vis absorption spectra (Fig. 3b) [42]. All the absorption peaks of these Ag NPs are located at *ca*. 420 nm and shifted to the higher energy. The results are assigned to the characteristic surface plasmon resonance of spherical Ag NPs and the size of as-synthesized products is increasing with the decrease in the amount of PVP [43, 44]. The width of each plasmon is related to the size distribution of the NPs [45]. Obviously, the bandwidth of absorption peak is gradually shifted to broader one, which is further demonstrated that the size distribution of Ag NPs becomes broader with the increased mass ratio of AgNO_3_ and PVP. Furthermore, the corresponding photographs of the prepared Ag NPs (S1 to S6) are presented in the insert of Fig. 3b. The color of the solutions from S1 to S5 gradually becomes darker, because of the different size distribution of high-yielded Ag NPs. However, the color of S6 is nearly transparent one, implying that the yield of prepared Ag NPs is low. The results reveal that PVP is not only served as capping agent against aggregation but also play a significant role of reducing agent because of abundant of hydroxyl groups in PVP [46]. Taking into account the yield of Ag NPs in this synthesis process, the low-yielded sample S6 is not used for further application of conductive inks.

**Fig. 4:**
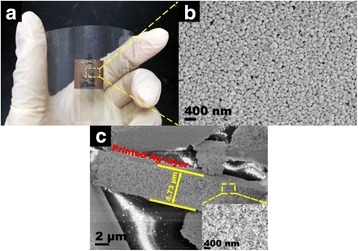
**a** Photograph of Ag NPs deposited on substrate when drying at room temperature without sintering treatment. **b** Top-view high-magnification SEM image of the Ag layer surface. **c** Cross-section SEM image of Ag pattern; *insert* is partial enlarged detail of cross section SEM image

Figure 4a shows the photograph of deposited Ag layers on PET without sintering, and the surface is flat and the color is deep. However, the color of sample presents white after sintered at 160 °C for 30 min (Additional file 1: Figure S1). There are no obvious detached fragments or cracks on the pattern found under bending, indicating high flexibility and mechanical toughness of the printed Ag patterns. The representative top-view SEM image of this Ag patterns is shown in Fig. 4b, and the spherical Ag NPs are accumulated in random. As shown in Fig. 4c, the thickness of the Ag layer is 4.73 μm.

**Fig. 5:**
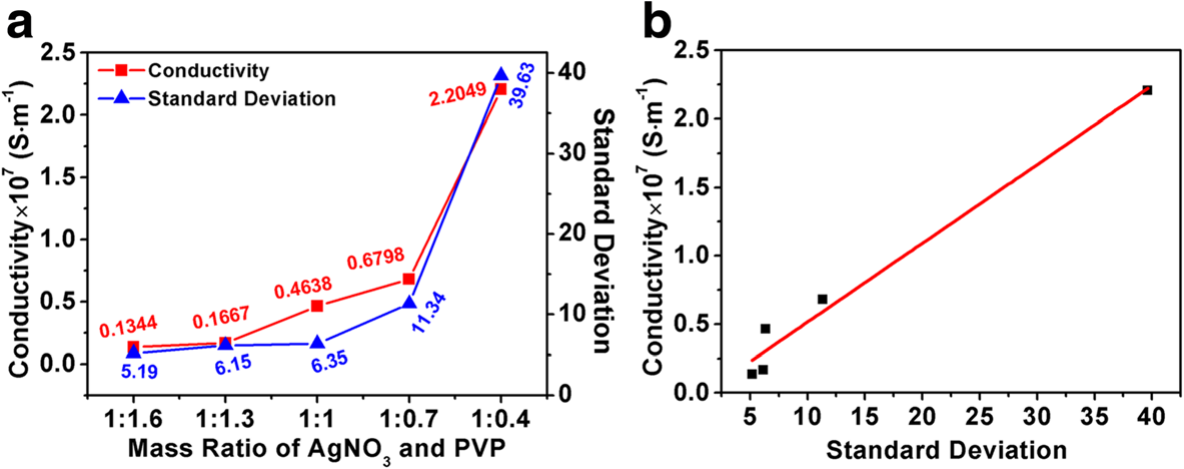
**a** Electrical conductivity of different products after sintering at 160 °C for 30 min and the standard deviation of different products with different size distribution. **b** The fitting curve of the conductivity and particle size standard deviation (*R* ^2^ = 0.97265)

As shown in Fig. 5a, the electronic properties of printed patterns by the aforementioned Ag NPs after sintering (at 160 °C for 30 min) are measured by the four-point probe resistance tester. Obviously, S5 exhibits the highest electrical conductivity and the value is 2.2049 × 10^7^ S · m^−1^. And the results reveal that the conductivity values are increased when the PVP concentration is decreased. Interestingly, it is noteworthy that a similar trend between standard deviation of particle size and the mass ratio of AgNO_3_ and PVP is also found. Therefore, a linear fitting curve of conductivity and standard deviation of particle size is plotted (Fig. 5b). The linear fitting results demonstrate that the conductivity of Ag inks is highly dependent on the size distribution of Ag NPs. The sample S5 possesses the maximum standard deviation because the concentration of PVP is decreased, resulting in S5 obtaining the highest conductivity due to the smaller Ag NPs that could fill the gap between the large particles. Thus, these Ag NPs can be more fully contacted with each other than other samples. Subsequently, the surface morphologies of all samples (sintering at 160 °C for 30 min) are investigated by SEM, and the results are shown in Additional file 1: Figure S2 (Supporting Information). As shown in Additional file 1: Figures S2a and S2b, the Ag NPs are independent from each other and a small proportion of Ag NPs begin to melt and connect together in samples S1 and S2, which bring the lower electrical conductivity. When the mass ratio of AgNO_3_ and PVP keep decreasing, the Ag NPs melt and connect more and more closely and finally format as a conductive network (Additional file 1: Figures S2c, S2d, and S2e). Obviously, the Ag NPs with a wider size distribution are beneficial to increase the conductivity, and the results are in agreement with Fig. 5. Therefore, sample S5 is selected as a candidate for further electronic property investigation.

**Fig. 6:**
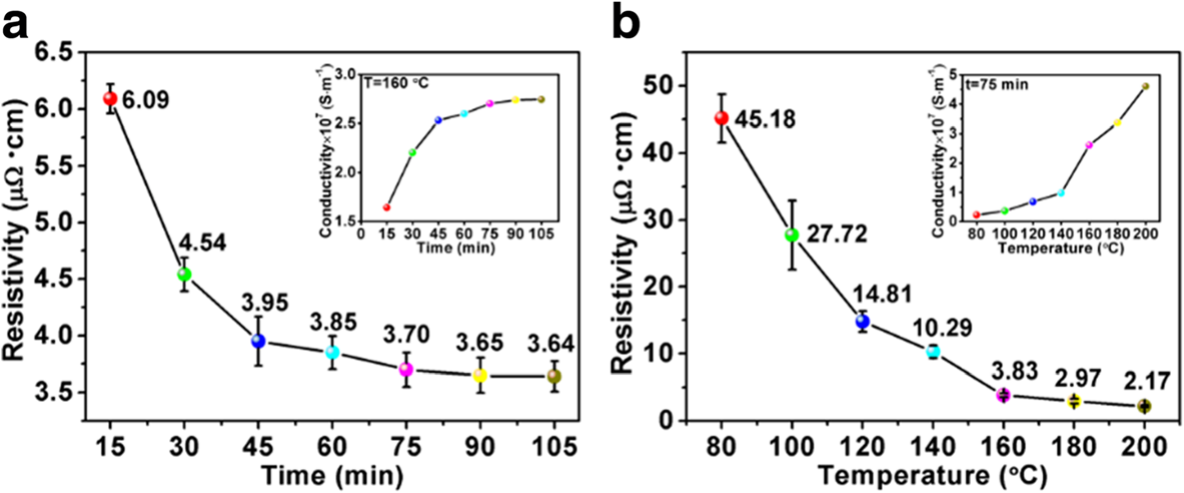
**a** The resistivity of printed patterns sintered at 160 °C for different time; inserted figure is the corresponding conductivity. **b** The resistivity of deposited patterns sintered at different temperatures for 75 min; inserted figure is the corresponding conductivity

The resistivity (or conductivity) of printed patterns is significantly influenced by the sintering time and temperature. Firstly, in order to investigate the influence of sintering time, the time-dependent resistivity of the pattern was calculated after sintering at 160 °C for different times (15–105 min), as shown in Fig. 6a. The resistivity value of the pattern is decreased rapidly before the sintering time of 75 min. Then, a steady resistivity is achieved after 75 min, and the conductivity is 2.7018 × 10^7^ S · m^−1^. According to Eq. 2, an inverse trend of the conductivity of the sintered pattern is exhibited in the *insert* of Fig. 6a. Subsequently, the influence of the sintering temperature on the electronic performance is further investigated, and the sintering time is fixed at 75 min. As shown in Fig. 6b, the resistivity of sintered pattern is decreased with the elevating sintering temperature. Furthermore, the resistivity is decreased rapidly from 45.18 to 3.83 μΩ · cm when the sintering temperature was increased from 80 to 160 °C. When the sintering temperature was further increased to 200 °C, a lower resistivity of 2.17 μΩ · cm is obtained, which is very close to the bulk silver (1.58 μΩ · cm). To avoid the destruction of PET substrates, the sintering parameters are selected at 160 °C for 75 min, and the average resistivity of obtained patterns is relative low (3.83 μΩ · cm), which is about 40 % of the bulk silver.

**Fig. 7:**
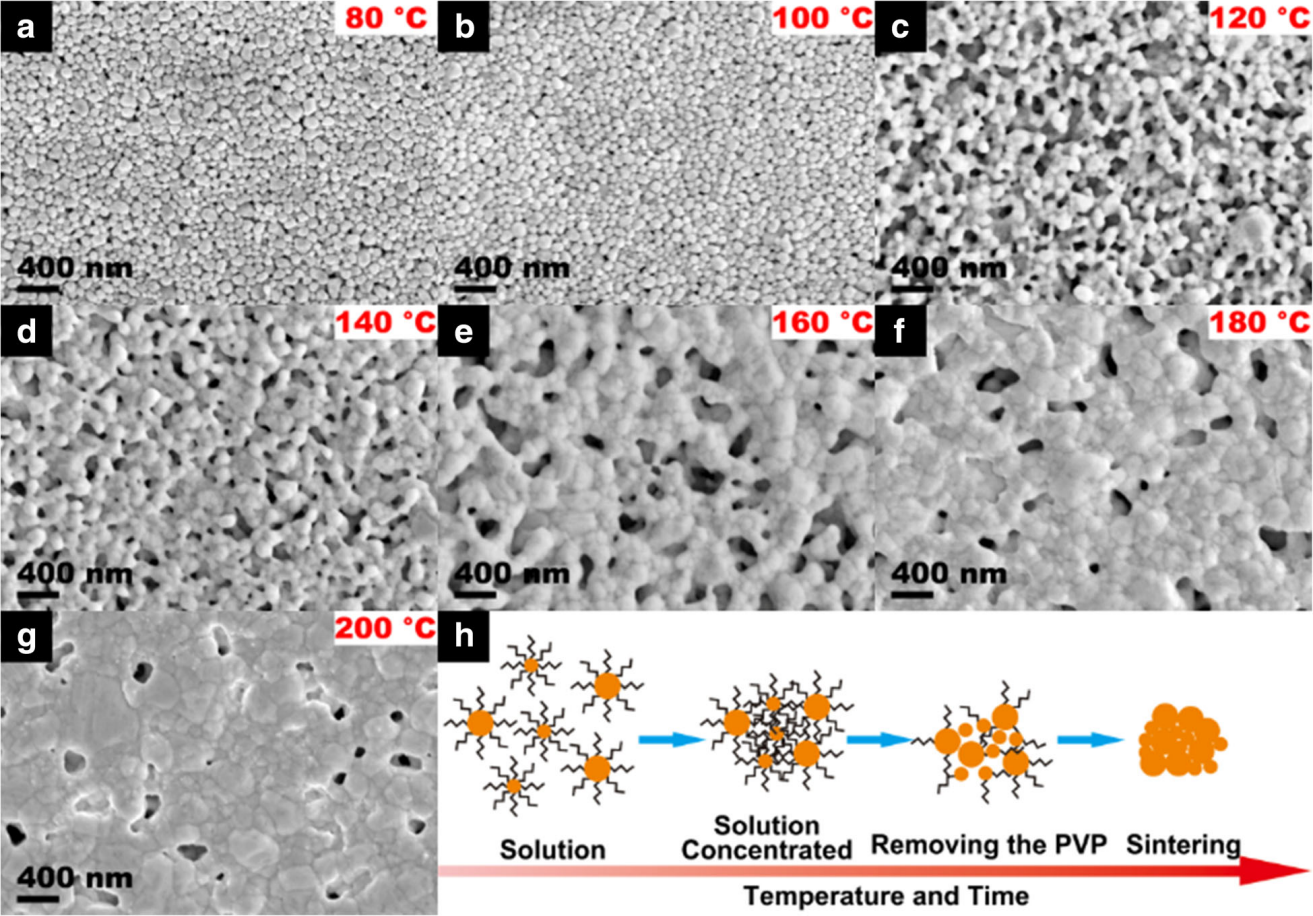
SEM images of silver patterns after sintering on PET substrates at different sintering temperatures for 75 min. **a** 80 °C. **b** 100 °C. **c** 120 °C. **d** 140 °C. **e** 160 °C. **f** 180 °C. **g** 200 °C. **h** The schematic illustration of the sintering mechanism

We used the SEM to study the reason of sintering time and temperature-dependent electronic performances of printed samples. Additional file 1: Figure S3 (Supporting Information) shows the effect of sintered time on the morphology of patterns. Aggregated Ag NPs with distributed pores are performed at initial sintering time of 15 to 45 min. Then, when the sintered time was increased to 75 min, the aggregated Ag NPs are inclined to merge under the driving force of diffusion, resulting in the distributed pores disappearing and the Ag NPs melting into a conductive network. Such merge among Ag NPs for compacted layer of printed patterns is continuously performed when the sintering time was further increased to 105 min. As a result, higher conductivity of printed patterns is realized at long sintering time of 105 min, as consistent with the results in Fig. 6a. Then, the influence of sintering temperature on the morphology of printed patterns is presented in Fig. 7. The lower sintering temperatures of 80 and 100 °C have no effect on the morphology of printed patterns because of the protection effect of the remained organic molecules, resulting in lower electrical conductivity (Fig. 7a, b) [47]. When the sintering temperature was increased to 120 and 140 °C, the aggregated Ag NPs with numerous distributed pores are presented gradually (Fig. 7c, d). However, the density of distributed pores is gradually decreased when the sintering temperature was further increased, resulting in a smooth compacted layer of printed pattern for excellent conductivity performance (Fig. 7e–g). A proposed schematic illustration of the sintering process is displayed in Fig. 7h. Silver particles uniformly disperse in a solvent for the presence of remaining organic stabilizing agents of the inks between the particles at ambient temperature. At the lower sintering temperature or short sintering time, the Ag inks are concentrated. With the increase of sintering temperature or sintering time, the PVP starts to remove from the surface of Ag NPs. The Ag NPs with large size begin to melt and contact with each other to form conductive paths, and the small Ag NPs pack into the gap between the large ones. Finally, at higher sintering temperature or longer sintering time, the Ag NPs sinter together to create 3D conductive networks [48]. The mechanical stability of printed patterns is tested by the bending test. The printed patterns were bending over 90° and repeated the bending movement for many times. As shown in Additional file 1: Figure S4 (Supporting Information), the resistivity of the printed patterns after bending (every 10 cycles) was measured. The resistivity of printed patterns increased slightly with the bending cycles, which indicates good mechanical stability and adhesion.

**Fig. 8:**
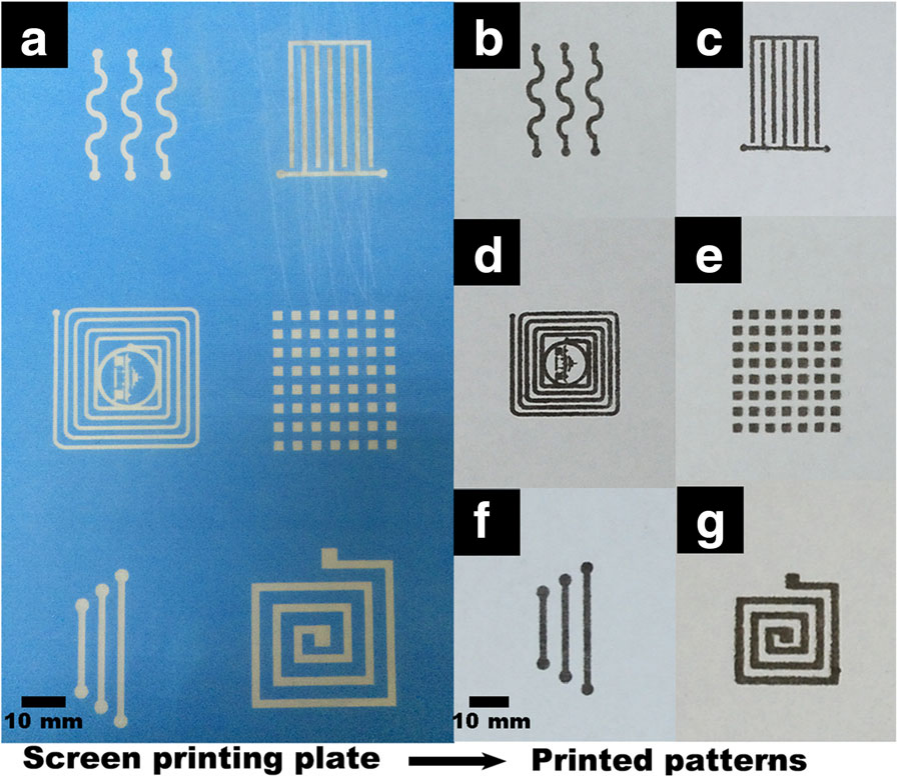
**a** Photographs of the part of designed screen printing plate and the corresponding printed conductive patterns on the paper by using the as-prepared Ag NPs through screen printing (**b**, **c**, **d**, **e**, **f**, **g**)

The paper is also an important substrate for flexible printed electronics, which has received broad interest because of its huge commercial value [48, 49]. The Ag inks are also employed for different patterns on a paper-based flexible substrate through screen printing. The image of the designed screen printing plate is displayed in Additional file 1: Figure S5 (Supporting Information). Then, the photographs of the corresponding printed patterns are shown in Fig. 8, indicating that as-prepared Ag inks possess an excellent printability for flexible paper and PET substrates. The photographs of the corresponding printed patterns are shown in Fig. 8. These patterns were sintered at 100 °C for 75 min, and the average resistivity is 19.17 μΩ · cm. The as-prepared Ag inks also possess an excellent printability for flexible paper.

## Conclusions

In this study, through a facile and one-step polyol method to synthesize the Ag NPs with different size distribution, the results of broad size distribution that contribute to the improvement of conductivity were found. The optimal electrical resistivity of 3.83 μΩ · cm was achieved by heat treatment at 160 °C for 75 min, and the electrical resistivity of as-obtained Ag inks is very close to the electrical resistivity of the bulk silver. The present results suggest possible applications in flexible printed electronics.

## Additional File

Additional file 1: Supporting information. (DOCX 3669 kb)
