# Supplementary material for: Preparing of Highly Conductive Patterns on Flexible Substrates by Screen Printing of Silver Nanoparticles with Different Size Distribution
Source: Nanoscale Res Lett. 2016 Sep 20;11:412. doi: 10.1186/s11671-016-1640-1 (PMC5028354; doi:10.1186/s11671-016-1640-1)
Supplement: Additional file 1: — Supporting information. (DOCX 3669 kb) [file 11671_2016_1640_MOESM1_ESM.docx]

Supporting information

Preparing of Highly Conductive Patterns on Flexible Substrates by Screen Printing of Silver Nanoparticles with Different Size Distribution

*Jin Ding^1^* *, Jun Liu^2^, Qingyong Tian^2^, Zhaohui Wu^1^, Weijing Yao^1^, Zhigao Dai^1^, Li Liu^1^, Wei Wu^1,3^^[[1]](#footnote-1)^**

^1^ Laboratory of Printable Functional Nanomaterials and Printed Electronics, School of Printing and Packaging, Wuhan University, Wuhan 430072, P. R. China

^2^ Key Laboratory of Artificial Micro- and Nano-structures of Ministry of Education, School of Physics and Technology, Wuhan University, Wuhan 430072, P. R. China

^3^ Shenzhen Research Institute of Wuhan University, Shenzhen 518057, P. R. China


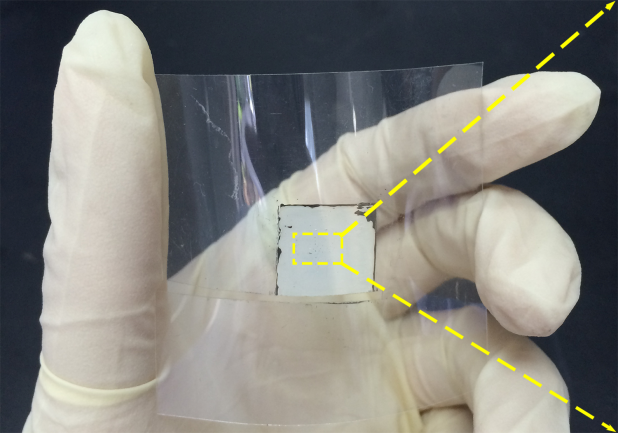


**Figure S1.** Photograph of Ag NPs deposited on substrate when drying at 160 °C for 30 min


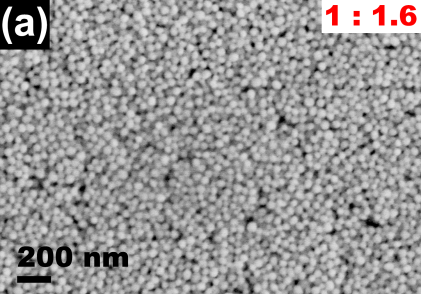

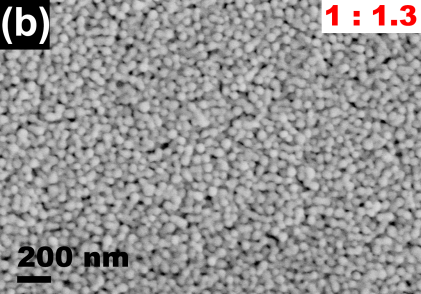

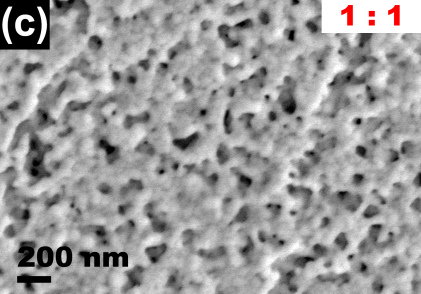

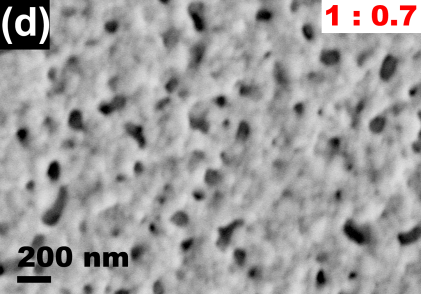

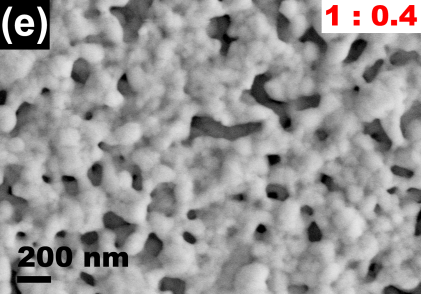


**Figure S2.** (a) SEM images of the as-obtained Ag patterns with different mass ratio of AgNO_3_ and PVP: (a) 1:1.6, (b) 1:1.3, (c) 1:1, (d) 1:0.7, (e) 1:0.4 after sintering at 160 °C for 30 min.

**
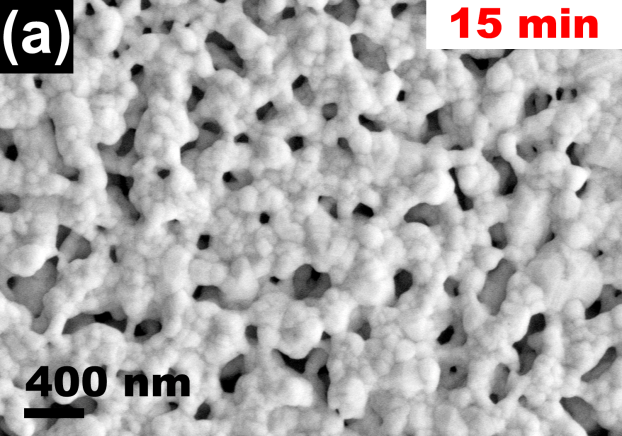

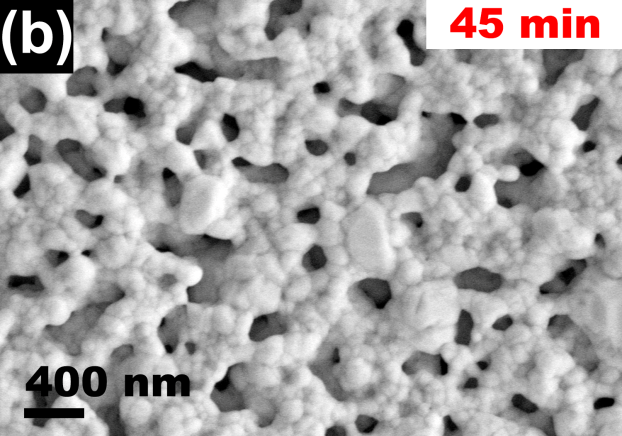

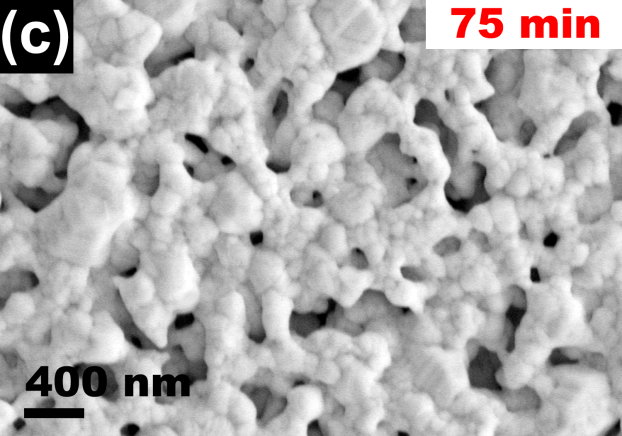

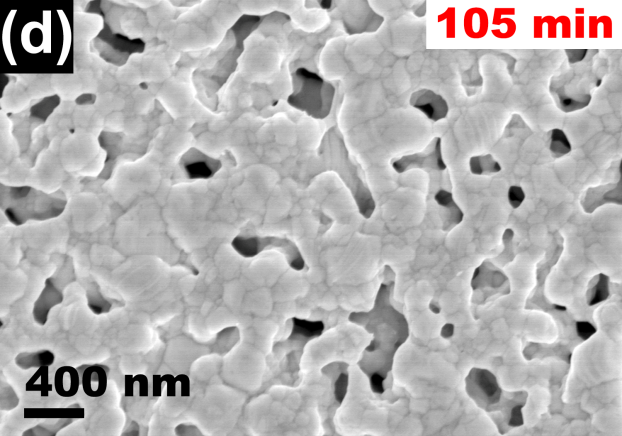
**

**Figure S3.** SEM images of silver patterns after sintering at 160 °C for different time: (a) 15 min, (b) 45 min, (c) 75 min, (d) 105 min.


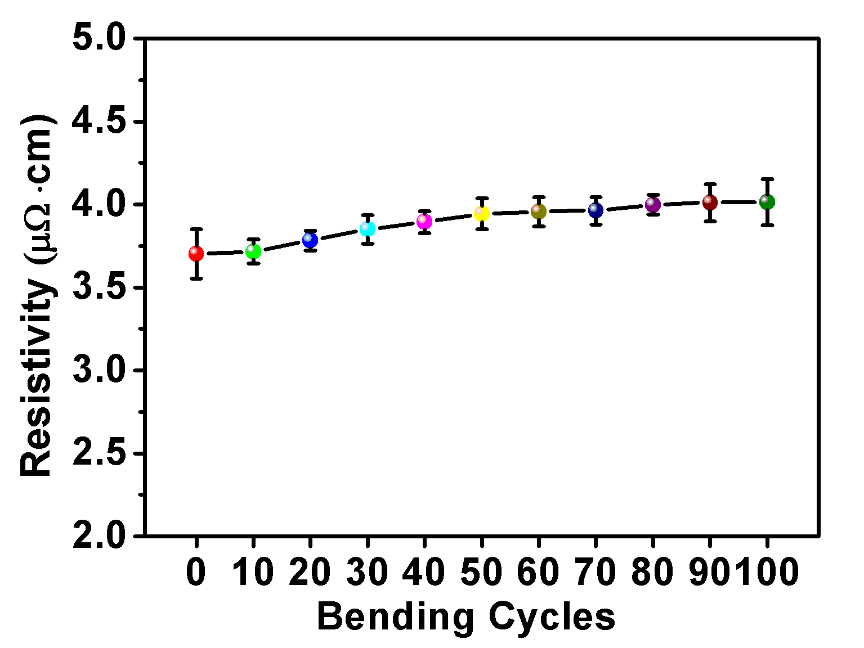


**Figure S4** The resistivity of printed patterns on PET substrate sintered at 160 °C for 75 min after repeated bending cycles.


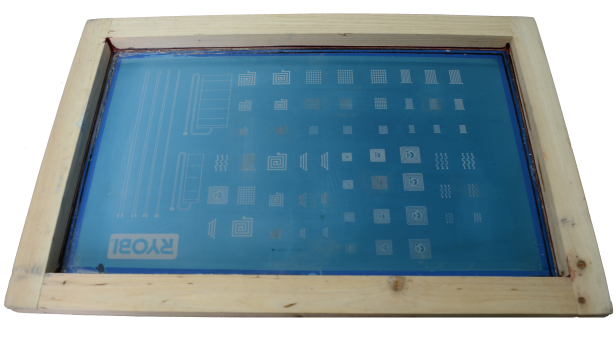


**Figure S5** The image of screen printing plate.

1. *To whom correspondence should be addressed. Tel: +86-27-68778529. Fax: +86-27-68778433. E-mail: [weiwu@whu.edu.cn](mailto:weiwu@whu.edu.cn) (W. Wu) [↑](#footnote-ref-1)
